# Development of High-Fibre and Low-FODMAP Crackers

**DOI:** 10.3390/foods11172577

**Published:** 2022-08-25

**Authors:** Kristina Radoš, Nikolina Čukelj Mustač, Katarina Varga, Saša Drakula, Bojana Voučko, Duška Ćurić, Dubravka Novotni

**Affiliations:** Faculty of Food Technology and Biotechnology, University of Zagreb, Pierottijeva 6, 10000 Zagreb, Croatia

**Keywords:** wholemeal formulations, low fructan, sensory analysis, ready-to-eat, oilseeds, spices, sourdough, gluten-free

## Abstract

Since there are no products in the European market labelled as low-FODMAP (low in fermentable oligosaccharides, disaccharides, monosaccharides, and polyols), patients with irritable bowel syndrome and non-celiac wheat sensitivity often consume gluten-free products. These naturally contain little FODMAP, but have poorer sensory properties and lower nutritional value. This study aimed to develop sensory attractive crackers with high-fibre and low-FODMAP content. Various gluten-free flours (wholemeal buckwheat and millet, white maize), pumpkin seed meal, chia seeds, flax seeds, rice protein, sweet potato, sourdough, and spices were used to develop nine formulations. Using a nine-point hedonic scale and ranking test, four best-scored products were selected for which descriptive sensory analysis was performed and nutritional value and fructan content were determined. Crackers made from maize and millet flour mixtures (ratio 1:2.5) with sourdough and with chia or flax seed addition were rated highest for overall impression (8.2 and 7.0, respectively). Generally, high-fibre content, hardness, chewiness, dark colour, and bitterness lower the acceptability of crackers, but the addition of spices and sourdough can improve their acceptability and marketability. The crackers could be labelled as “gluten-free”, “low-FODMAP” (<0.12 g/100 g), “naturally high-fibre” (7–10 g/100 g of which 17–23% are soluble), and “high in protein” (24–26 g/100 g).

## 1. Introduction

Non-celiac wheat sensitivity (NCWS) and irritable bowel syndrome (IBS) are the growing metabolic disorders for which an adapted and individualised diet is usually recommended as an integral part of the treatment. Low fermentable oligosaccharide, disaccharide, monosaccharide, and polyol (FODMAP) diets show the best effect in alleviating the symptoms of IBS and NCWS. FODMAPs are important natural prebiotics and their intake affects the structure and function of the gut microbiota. However, they are also characterised by poor absorption in the small intestine, fermentability, and high osmotic activity, which can cause digestive problems in sensitive individuals. In fact, reducing FODMAPs is cited as more likely to improve symptoms in NCWS than eliminating gluten-containing products [1].

An important principle of the low-FODMAP diet is the replacement of intolerable high-FODMAP foods with nutritionally equivalent low-FODMAP foods. This is a major challenge as avoiding FODMAP can easily lead to low intake of dietary fibre and micronutrients, which, if not compensated by suitable alternative sources of fibre, can lead to undesirable changes in the gut microbiota [2]. Therefore, more attention should be paid to the development of tasty functional ready-to-eat products with balanced nutrient composition but with low-FODMAP content [3]. Ready-to-eat products, such as snacks, have become an important part of the diet in the fast-paced modern world. Commonly, cereal snacks are high in saturated fats, simple sugars, or sodium [4], while low in fibre. However, for people suffering from NCWS, adequate intake of fibre (21 to 38 g per day) is crucial as chronic constipation is a common symptom [5]. The use of wholemeal cereals, oilseeds, and by-products from oil processing holds great potential for the development of functional snacks. Pumpkin, flax, and chia seeds are characterised by high biological and nutritional value due to their high protein, fibre, and unsaturated fatty acid content, and are also a rich source of minerals, vitamins, carotenoids, and bioactive compounds [6]. Pumpkin seed meal is the main by-product in the production of pumpkin oil and is a source of proteins and unsaturated fatty acids, but it is also a rich treasury of carotenoids, bioactive compounds, vitamins, and minerals [7,8]. Wholemeal cereals contain a high amount of dietary fibre but can contain FODMAPs such as fructans including fructooligosaccharides (FOS) and galactooligosaccharides (GOS), which are mainly concentrated in the bran [9].

Thus, there are several strategies to produce low-FODMAP products. They can be produced either by selecting low-FODMAP ingredients in the product formulations or by using biotechnological tools to degrade FODMAP during the production process (e.g., sourdough, yeast fermentation, and the application of enzymes) [9,10]. Wholemeal flours from naturally gluten-free grains offer a high content of fibre while a limited amount of FODMAP [3]. The main benefits of using sourdough in wholemeal bakery products are flavour development together with extended shelf life, reduced glycaemic index, and increased bioavailability of mineral and bioactive compounds [11]. Moreover, Loponen and Gänzle [10] showed that the use of sourdough in the production of wholemeal bread significantly reduced the content of FODMAP in bread without affecting the content of slowly fermented and well-tolerated dietary fibre. Recently, Habuš et al. [9] showed that sourdough fermentation of amaranth and wheat bran decreased fructan content by up to 93%. Regardless of the method used, low-FODMAP alternatives should have the same or better nutritional and sensory quality than conventional food products. Thus, there is need to investigate these approaches in the development of a low-FODMAP snack.

In the development of a new product, consumer acceptance continues to be the most important factor, while sensory analysis is the gold standard in assessing product acceptance. Consumers are attracted to good sensory attributes, and therefore, sensory methods are an important and integral tool that should be used in the new product development process [12]. Sensory evaluation of a product makes it possible to obtain more information about the product being analysed, its quality, and to review the factors that influence its acceptance by consumers, which facilitates work on improving the quality of the product or its reformulation [13].

Currently, very few FODMAP-labelled products are available on the European market, due to the lack of definitions and EU legislation for FODMAPs. Low-FODMAP foods can be found among gluten-free products, but these are rarely sensory attractive and have low nutritional value [3]. A good example of FODMAP awareness can be found in Australia, where products are certified as low-FODMAP by Monash University and/or can go through the FODMAP Friendly Certification Programme (FODMAP everyday. Available online: https://www.fodmapeveryday.com/ accessed on 14 August 2022). The criteria that must be met for certification are not publicly available, and each product is evaluated individually. However, in general, products or formulations must not contain added FODMAPs, including fructo-oligosaccharides, inulin, and polyols (except sorbitol and mannitol), maltitol, xylitol, erythritol, lactitol, and isomalt. Among the certified products, there are only two cracker products labelled as “Low-FODMAP”.

The aim of this study was to investigate the possibility of using different gluten-free wholemeal cereals, oilseeds, plant protein, sweet potato, sourdough, and spices in the development of nutritionally enhanced and sensory attractive crackers for consumers with IBS and NCWS. The first phase was the development of nine cracker formulations with high fibre but low fructan content; and the second phase involved selecting the four best rated crackers and evaluating them for their sensory description, nutritional value, and fructan content. The chemical composition of the developed crackers was compared with the chemical composition of two similar products available on the market.

## 2. Materials and Methods

### 2.1. Raw Materials

The cracker formulations contained combinations of different gluten-free raw materials, as these usually contain small amounts of FODMAP. The raw materials were white maize flour, wholemeal buckwheat, and millet flour (all three from Pukanić Mill Ltd., Velika Gorica, Croatia) (median particle size D(50) ~300 µm), sweet potato puree (dm-drogerie markt GmbH + Co. KG, Karlsruhe, Germany), flax seeds (SME Osijek, Osijek, Croatia), chia seeds (Nutrigold, EU), pumpkin seed meal (D(50) ~500 µm, PoljoPosavec Ltd., Dunjkovec, Croatia), and rice protein (Nutrigold, EU). The formulations also contained fat spread consisting of 59% vegetable oil (Omegol, Zvijezda Ltd., Zagreb, Croatia), olive oil (Olitalia, Forli, Italy), LIVENDO^TM^ LV1 starter (Lessafre Inc., Paris, France), spices (dill, pepper, tumeric, chives, wild garlic, Mediterranean seasoning mix, garlic, rosemary, chili, thyme, basil, and parsley), dry yeast (Lesaffre Adriatic Inc., Prigorje Brdovečko, Croatia), salt, and tap water. Raw materials were used without milling except chia and flax seeds which were ground in a coffee grinder until a particle size of <500 µm was achieved, and sieved using a laboratory rotary vibration sieve. Median particle size was determined by laser diffraction on a Mastersizer 2000 device (Malvern Panalytical, Malvern, UK) in combination with a dry dispersion unit (Scirocco 2000) at a pressure of *p* = 1 bar and a cell supply rate of 60%, in three replicates. The proximate composition taken from producers’ labels of main ingredients is shown in Table 1. The proximate composition of our crackers was compared with commercially available certified low-FODMAP crackers (San-J Tamari Black Sesame and San-J Tamari Brown Sesame) (FODMAP everyday. Available online: https://www.fodmapeveryday.com/product-category/collections/low-fodmap-certified-brands/ (accessed on 14 August 2022)). The ingredients in the one of the commercial crackers were black sesame, potato starch, brown rice, soy sauce, water, dextrin and salt, while the ingredients in the other commercial cracker were sesame, brown rice, rice flour and sweet potato starch and soy sauce.

### 2.2. Methods

#### 2.2.1. Determination of Moisture, Ash, Carbohydrates, Fats, Proteins, and Fibre Contents

The chemical composition of the four best-rated crackers was determined by standard AACC methods [14] in two replicates. The moisture content was determined according to the AACC method 62-05; the ash content was determined by incineration at 550 °C according to the AACC method 08-01. The amount of protein was determined using the Kjeldahl method, according to the AACC method 46-12. The fat content was determined using the Soxhlet method according to the AACC method 30-25.01, and fatty acids were determined by gas chromatography of their methyl esters as previously described by Balbino et al. [15]. The share of total carbohydrates was determined by subtracting the mass of water, fat, protein, and ash from the tested amount of food (100 g). Dietary fibre (insoluble and soluble) content was determined using an enzymatic kit (Integrated Total Dietary Fibre Assay kit, Megazyme, Wicklow, Ireland). Since the manufacturer’s labels did not provide information on the amount of soluble fibre or fibre at all, as well as information on ash and fructan content (important data in the development of products for IBS and NCWS patients), these were also determined in flours as well as in rice protein, chia seeds, flax seeds, and pumpkin seed meal. The energy values of crackers were calculated by multiplying the factor values, i.e., 1 g of crude protein or carbohydrate provides 4 kcal/16 kJ of energy, 1 g of crude fat provides 9 kcal/36 kJ of energy, and 1 g of fibre provides 2 kcal/8 kJ.

#### 2.2.2. Determination of the FODMAP Content

Determination of fructan and GOS content in white maize flour, millet, buckwheat, rice protein, chia seeds, flax seeds, pumpkin seed meal, and four selected crackers was performed according to the AOAC method 999.03, as previously described by Habuš et al. [9], without adding enzyme α-galactosidase. A Fructan Assay Megazyme kit (Megazyme, Wicklow, Ireland) and Mega-CalcTM calculator were used to calculate the fructan content of the samples based on the measured absorbances at 410 nm (Analytik Jena, SPECORD 50 PLUS, Jena, Germany).

#### 2.2.3. Development of Cracker Formulations

In the first phase, nine formulations of crackers (Table 2) were developed based on the proximate composition of raw materials. Maize, proso millet, and buckwheat flour were chosen as the main raw materials. We wanted to avoid using rice flour as a well-known and commonly used ingredient in gluten-free products, as can be seen in the example of commercially available low-FODMAP crackers. Based on our previous successful use of millet, corn, and buckwheat flours in bread development (to be published), the above flours were selected for the cracker formulations. The proportions of flours were determined by preliminary experiments, while other ingredients were dosed to meet nutritional recommendations. The formulations were developed to be high in fibre which, according to EU regulation 1169/2011/EU, meant that the product contained at least 6 g of fibre per 100 g, but also satisfied the needs of patients with IBS and NCWS, since the latest recommendations are to include 25–35 g fibre per day or ≥14.6 g/1000 kcal per day for adults [16].

#### 2.2.4. Preparation of Crackers

Eight of the nine types of crackers included the sourdough fermentation. To make sourdough, 20 g of flour mix (depending on the recipe), 35 mL of room temperature tap water, and 0.5% (flour basis) of LV1 starter were used. After fermentation in a lidded jar at 30 °C for 16 h, sourdough was added to the main dough mixture considering the amount of flour and water contained in the sourdough. The cracker F2 was made without sourdough using the “all-in” method. The cracker dough was prepared using a kitchen robot (EKM4000, Electrolux, Stockholm, Sweden) according to the modified AACC 10-50D method [14]. The method was used as a guide for formulations of crackers, while the water was adjusted in preliminary tests, taking into consideration the workability and stickiness of the dough. First, fat and sugar were mixed in a kitchen blender (slowly, one minute). Then, depending on the recipe, rice proteins, soaked chia seeds (soaked in twice the amount of water for 30 min at room temperature), flax seeds, pumpkin seed meal, salt, and spices were added and mixed slowly for three minutes. The next step was to add the sourdough (if included) whose pH value had previously been measured, the remaining water, and the sweet potato puree (stirred slowly for one minute, and then quickly for one minute). Finally, the flour was added and the dough was mixed slowly for 10 min. The prepared dough rested in the refrigerator for 30 min. Then, the dough was then rolled out manually on a board with thickness spacers to a thickness of three millimetres, cut out with a 6 cm diameter cutter, and punched. The crackers were baked in a deck oven (EBO 64-320 IS 600, Wiesheu GmbH, Großbottwar, Germany) at a temperature of 180 °C for 10 min on one side and 10 min on the other side, after which the crackers were turned again and baked for another 10 min. After baking, the crackers were left to cool for 30 min at room temperature.

#### 2.2.5. Sensory Analysis of Crackers

The sensory analysis of the crackers was carried out at the Faculty of Food Technology and Biotechnology at the University of Zagreb, Zagreb, Croatia. A panel of nine experienced assessors (aged 23 to 52 years) who specialised in cereal products was selected. The training programme for the sensory panel included the identification and description of attributes and the procedures using the response scale [17]. All of the samples were coded using three-digit numbers, and served with water to rinse the palate. First, a hedonic sensory analysis was performed, in which all of the nine samples were evaluated according to overall impression on a scale from 1 (“extremely dislike”) to 9 (“extremely like”). Then, a ranking test was performed in which the subjects ranked the samples from the most preferred (score of 1) to the least preferred (score of 9). The samples with an average score of more than 5.5 were selected. In the next step, the intensity of sensory properties of the selected crackers was evaluated on a scale from 0 (not perceived) to 10 (very intense) by descriptive sensory analysis, performed according to the standards ISO 13299:2003 and 6658:2017 [18,19]. Prior to the assessment, the panel selected relevant sensory attributes (Table 3). The scoring included appearance, odour, taste, flavour, and texture parameters. This was followed by a hedonic sensory analysis of each sensory property of the four selected samples (appearance, odour, taste and flavour, texture in the mouth, overall impression).

### 2.3. Statistical Analysis

Microsoft Office Excel 2016 and Statistica v.10 (StatSoft Inc., Tulsa, OK, USA) were used for the statistical data processing. The results are expressed as the mean value with standard deviation. Statistica was used to detect statistically significant differences between samples for which analysis of variance (ANOVA) was performed with the Tukey post hoc test, with a value of *p* < 0.05 set as the limit of statistical significance.

## 3. Results and Discussion

### 3.1. Chemical Composition of Raw Materials and Fructan Content

Since there is a limited number of studies dealing with FODMAP content in wholemeal cereal and oilseed flours, we determined the content of fructan in the flours used (Table 4). White maize flour contained the least fructan, while fructan in buckwheat flour was below the detection limit. Knudsen et al. [20] determined the fructan content of whole maize flour to be 0.5 g/100 g sample dry matter. A low fructan content was also found in millet flour. This was consistent with the findings of Ispiryan et al. [3], who found that millet, buckwheat, and oats, which are common raw materials for gluten-free products, have fructan contents of less than 0.1 g/100 g sample dry matter, while their GOS content is low to moderate. Nevertheless, buckwheat, which is currently on the list of low-FODMAP cereals, contains the oligosaccharide phagopyritol, which is the most soluble carbohydrate. As its structure is similar to that of GOS, it potentially negatively affects the symptoms of IBS, which requires further research [3]. A low fructan content was determined in the rice protein, while the highest concentrations were found in pumpkin seed meal and flax seeds. The content of FODMAP in foods depends on the applied production process. For example, if soluble carbohydrates, which include GOS, are not removed during processing, the product will have a higher content of FODMAP [3]. Walnuts, peanuts, and pumpkin seeds, contrary to cashews and pistachios, are examples of nuts and seeds with low-FODMAP content [21]. The limit for oligosaccharides (total fructans and GOS) is 0.3 g in a standard serving of whole grain products, nuts, legumes, and seeds [22]. Although the FODMAP content in a particular food may be slightly higher, the total amount absorbed by the body is important and should not exceed the cut-off value of 0.3 g per serving (30 g snack = 1 serving). A serving of two tablespoons of chia seeds contains sufficiently low FODMAP content that it should be tolerated by most people with IBS [23]. In this research, flax seed has been found to contain about twice the fructose content of chia seeds. Thus, eating one tablespoon of flax seed is considered to be a low source of FODMAP, while larger amounts should be avoided [23].

Total dietary fibre content in the raw materials used ranged from 2 to 38 g per 100 g of raw material (Table 4). Chia seeds, flax seeds, and pumpkin seed meal are rich sources of fibre with pumpkin seed meal having the highest proportion of soluble fibre among them (46%). Among cereal flours, millet flour was dominant in both total and soluble (50%) fibre content. The most recent clinical guidelines on the management of IBS consider soluble fibre as a reasonable first line therapy for IBS patients with symptoms. In contrast, products containing insoluble fibre, particularly wheat bran, do not appear to be useful in treating IBS symptoms [23]. Indeed, unlike insoluble, soluble fibre significantly improved the global assessment of IBS symptoms as compared with placebo.

According to Coskuner and Karababa [24], flax seeds on average contain 30–40% fat, 20–25% protein, 20–28% total fibre, 4–8% moisture content, and 3–4% ash. The chemical composition of flax seeds used in this study was similar to the above values. As expected, white maize flour was low in fibre, while rice protein was a source of fibre [25] Plant proteins are often used to increase the nutritional value of gluten-free products or to improve the rheological properties of the dough. Similarly, chia seeds improve the rheology of the gluten-free dough due to their high ability to absorb water, up to 15 times their mass, which leads to the formation of a gel [26].

The highest mineral content was found in pumpkin seed meal, followed by chia and flax seeds. Comparing the mineral contents of the flours, buckwheat flour is dominant in terms of minerals. Although the addition of buckwheat flour has a negative impact on the sensory acceptance of the product due to its characteristic taste, it contributes to the nutritional value of the product [27]. The lowest mineral content was found in white maize flour. In the gastrointestinal tract, vitamins and minerals are important for nutrient absorption, gut motility, modulation of the human gut microbiome, and other functions [28]. In research by Roth et al. [29], intake of micronutrients by IBS patients was lower than recommended, which was associated with gastrointestinal and extraintestinal symptoms, as well as with fatigue.

### 3.2. Results of Sensory Analysis

#### 3.2.1. Acceptability and Preference of Nine Crackers

**Figure 1:**
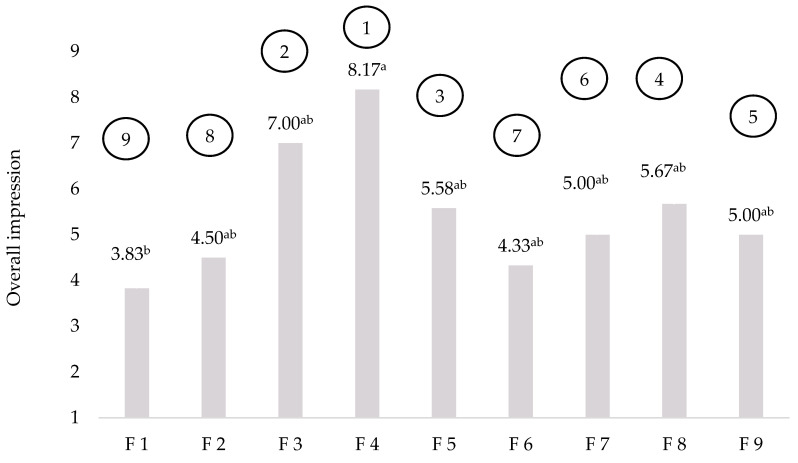
Results of the hedonic sensory analysis of the overall impression and the ranking test (in circles) of nine different cracker formulations (samples among the column marked with different letters are statistically significantly different (*p* < 0.05)).

A hedonic sensory analysis and ranking test were used to determine the general acceptability and preference of crackers produced according to nine different formulations (Table 2). Only four crackers had hedonic scores higher than 5.5 (common cut-off point for product marketability [30]) and were chosen for further chemical and sensory analyses. Furthermore, crackers were targeted to contain about 15 g of dietary fibre per 1000 kcal, which was achieved for selected crackers. Crackers prepared according to F3 and F4 formulations achieved the highest average scores for overall impression (Figure 1). The cracker made according to the F3 formulation was “moderately liked”, while cracker F4 was “liked very much”. They were followed by crackers F5 and F8 “neither liked nor disliked”. This neutral liking is probably related to the fact that crackers F5 and F8 contained buckwheat flour which has specific sensory properties. On the contrary, Sedej et al. [31] observed no significant differences in sensory quality of wholegrain buckwheat crackers as compared with wheat crackers. The overall impression of the other crackers was rated as “slightly dislike”.

The preference rank order is also presented in Figure 1 and is consistent with the results of the hedonic sensory analysis. In addition, a Friedman’s ANOVA did not find statistically significant differences in the ranking of the samples (Q = 0.043, *p* = 0.930).

#### 3.2.2. Sensory Profile of Four Best-Rated Crackers

**Figure 2:**
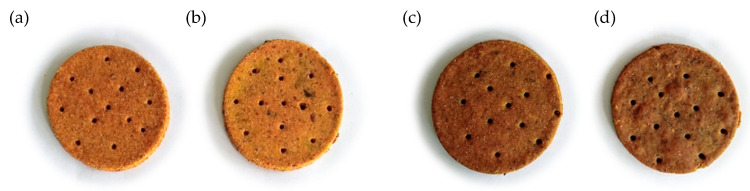
The appearance of four best-rated crackers: (**a**) F3; (**b**) F4; (**c**) F5; (**d**) F8.

**Figure 3:**
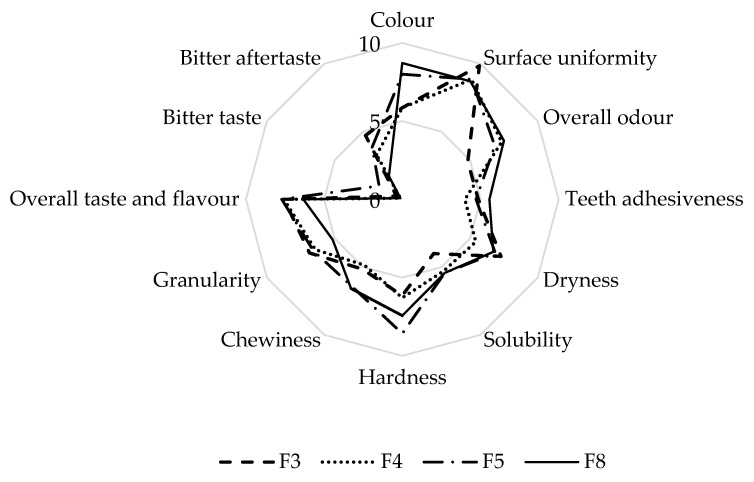
Intensity of sensory attributes of four best-rated crackers in the descriptive sensory analysis.

**Figure 4:**
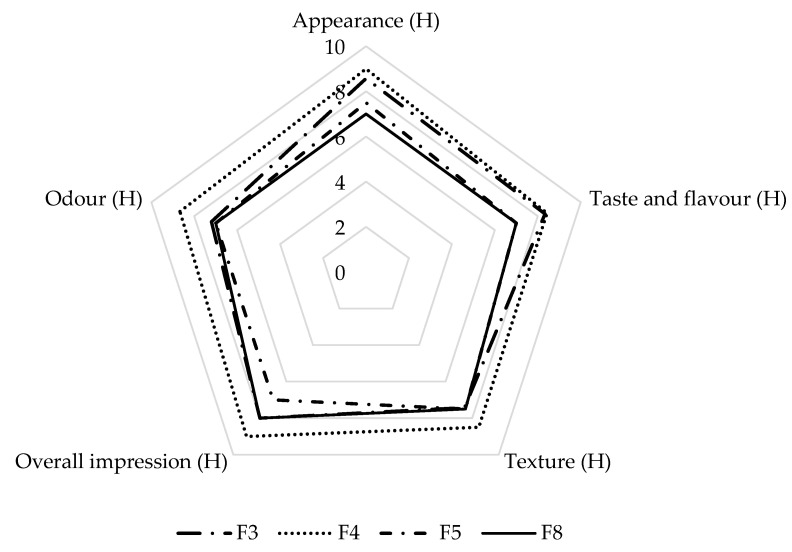
Acceptability of appearance, flavour, taste, odour, textural characteristics, and overall impression of four best-rated crackers in the hedonic sensory analysis.

The results of the descriptive and hedonic sensory analyses of four best-rated crackers (Figure 2) are shown in Figure 3 and Figure 4, respectively.

There was a statistically significant difference (*p* = 0.045) between the colour intensity of crackers F5 and F8 with an average value ≥8 as compared with the lighter F3 and F4, which had an average value of 5.9 for colour (Figure 3). The surface uniformity was similarly high among crackers.

The odour intensity of the crackers was strongly influenced by the spices used. In cracker F3, Mediterranean seasoning mix (a combination of garlic, rosemary, chilli, thyme, basil, and parsley) was used which resulted in the lowest scores for odour intensity (average 4.8). Chives were used in cracker F4, pepper and wild garlic were used in cracker F5, and turmeric and dill were used in cracker F8; crackers F4, F5, and F8 were rated similarly for odour intensity (6.8–7.5) which was higher than cracker F3.

A bitter taste was not detected in the crackers during consumption, in contrast to a well detected bitter aftertaste (in the range 1.7–4.7) in all of the four crackers. No significant (*p* > 0.05) difference was found between the crackers in terms of intensity of overall taste and flavour, chewiness, granularity, solubility, dryness, and teeth adhesiveness.

Cracker F5 was rated as the hardest (mean score 8.6), significantly harder than crackers F3 and F4 (*p* < 0.05) (mean scores 6.1 and 6.3), but not significantly harder than cracker F8 (mean score 7.4) (*p* > 0.05). The fat content in cracker F5 was lower than the fat content in the other three types of crackers, which most likely caused their higher hardness (Table 5). In addition to physical and chemical properties, fat has a positive effect on product viability by delaying the absorption of water from starch [32]. Therefore, the texture stability of cracker F5 could be the lowest.

The results of the hedonic sensory analysis of the individual properties of the four best-rated crackers are shown in Figure 4. Cracker F4 was best-liked in all examined properties (appearance, odour, taste and flavour, and texture, as well as overall), with average scores for each property above 8 (“like very much”), followed by cracker F3 with slightly lower average scores, but not significantly different from F4 (*p* > 0.05). The differences in the best-rated cracker F4 as compared with cracker F3 were the addition of flax seeds in the recipe and the use of chives, while in the cracker F3, chia seeds and Mediterranean seasoning mix was used.

Previous studies on the addition of flax seeds in biscuit production have shown that partial replacement of wheat flour with ground flax seeds (up to 12%) does not affect the physical and sensory properties of the product [33]. However, Čukelj et al. [6] achieved sensory acceptability of a multigrain flax seed biscuit similar to that of white wheat biscuits by using a suitable combination of cereal flour with ground flax seeds.

If we compare the results of the hedonic and descriptive sensory analyses, crackers F3 and F4 differed significantly only in intensity of overall odour, which was higher for cracker F4, suggesting that the better acceptance of cracker F4 was influenced by the spices used.

If we compare the results of the hedonic sensory analysis conducted on nine crackers with the sensory analysis conducted on four crackers in terms of overall impression, the best-rated crackers in both analyses were cracker F4, followed by cracker F3. However, in the second sensory analysis, crackers F5 were rated better than F8, while in first analysis it was vice versa.

Crackers F5 and F8 differed significantly from the better-rated crackers F3 and F4 (*p* < 0.05). Appearance, taste, and flavour, as well as texture in the mouth were rated better for cracker F5 than for cracker F8. Odour is the only sensory property that was rated better for cracker F8 than for cracker F5.

There are several factors that might influenced the better acceptance of cracker F5 as compared with cracker F8. First, several types of flour (maize, millet and buckwheat) were combined in cracker F5, while only buckwheat flour was used in cracker F8. In the research of Šimurina et al. [34], a wholegrain buckwheat cracker was rated better in terms of taste as compared with control wheat cracker, while in terms of odour, the control wheat cracker was rated better. In addition, chia seeds and pumpkin seed meal were included in our cracker F5, while flax seeds and sweet potato puree were used our cracker F8. We can conclude that combining buckwheat flour with other flours leads to better acceptability of crackers.

### 3.3. Nutritive Value, Fructan Content, and Labelling of Crackers

The moisture content in the final products was below 5 g in 100 g of sample, except for cracker F8 in which the moisture content was around 8% and could cause crackers to have a lower shelf life as compared with the other three crackers. Significantly higher content (*p* < 0.001) of minerals was found in crackers F5 and F8 as compared with crackers F3 and F4. The addition of pumpkin seed meal and buckwheat flour (Table 5) contributed to higher mineral content in crackers F5 and F8.

Crackers made according to the F8 formulation had the highest fat content, significantly differing from crackers F4 and F5 (*p* = 0.048 and 0.004, respectively), but not from cracker F3 (*p* = 0.183). In addition, all the crackers were low in saturated fat. In the F3, F4, and F5 formulations, 31–35% of the energy value originated from fats rich in unsaturated fatty acids.

Although significantly lower protein content was found in crackers F3 and F8 than in crackers F4 and F5, the differences were small. Pumpkin seed meal, which contains up to 65% protein [35], contributed to the higher protein content of cracker F5. Proteins were a source of 22–24% of the total energy value of crackers. Given that more than 20% of the energy value of crackers in four different recipes comes from protein, it could be labelled as “high in protein” [25]. Nutritional or health claims in the labelling, presentation and advertising of products while ensuring the effective functioning of the market also aim to ensure a high level of consumer protection.

In crackers F3, F4, and F5, the proportion of calories derived from carbohydrates was around 44%, while in cracker F8 it was slightly lower, around 40%. Thus, the stated calorie proportions derived from high-value proteins and carbohydrates were slightly lower than the recommended values of ~25–32% and 45–55%, respectively (Academy of Nutrition and Dietetics, Chicago, IL, USA). To achieve the target values for carbohydrates and proteins, the amount of fat should be reduced and the amount of proteins increased in our formulations. All the types of crackers were “high in fibre”, since they contained more than 15 g of fibre per 1000 kcal [25]. One serving of the crackers would contribute around 10% of the recommended daily intake (25 g). Nevertheless, crackers F4, F5, and F8 had higher proportions of soluble fibre (23–24%) as compared with cracker F3 with 17% soluble fibre (Table 5). Although insoluble fibre promotes regularity in a healthy digestive system, in patients with IBS, it can increase bloating, gas, cramping, etc., whereas soluble fibre helps improve IBS symptoms and slows the digestion of food [36]. Moreover, fructan levels in all four of the analysed crackers were below the cut-off value (Table 5). Thus, our crackers could be labelled as “low-FODMAP”, “naturally rich in fibre”, and “gluten-free”.

Considering the content of soluble fibre, crackers F4, F5, and F8 would be suitable for IBS patients, while all of the four crackers would be suitable for patients who suffer from NCWS and celiac disease. Hence, it can be observed that higher total dietary fibre content contrasts important descriptive sensory properties of crackers such as taste and flavour, while soluble fibre content contrasts undesirable descriptive texture attributes and bitter taste (Table 5 and Figure 4).

To the best of our knowledge, there are only two certified low-FODMAP crackers on the market, and they can be found in Australia (FODMAP everyday. Available online: https://www.fodmapeveryday.com/product-category/collections/low-fodmap-certified-brands/fodmap-friendly-low-fodmap-certified/?paged=8 (accessed on 14 August 2022)). The crackers contain about 21 g of fats, 55–60 g of carbohydrates, 4–7 g of fibre, 10–14 g of proteins, and approximately 450 kcal per 100 g of product. If we compare our crackers with those from the market, it is evident that they have less fat, much more proteins and fibre, and still have low-FODMAP content. The reason for this is that our crackers had more complex compositions and more nutritionally valuable ingredients such as wholemeal flours and oilseed flours. Furthermore, our crackers were prepared with sourdough which contributed to their flavour and taste, but also assured low-FODMAPs [9]. In particular, we observed about a 2.5-fold reduction in expected fructan level (almost 0.3 g/100 g) in cracker R5 containing pumpkin seed cake. In conclusion, oilseeds in a moderate amount can be safely used in the formulation of low-FODMAP crackers with sourdough.

## 4. Conclusions

The development of high-fibre low-FODMAP crackers requires the selection of a larger number of raw materials, as well as chemical and sensory evaluations. Wholemeal millet is a suitable raw material for such crackers because it is high in fibre, but more importantly, high in soluble fibre while low in FODMAPs. Furthermore, oilseed flours, as well as flours from by-products (pumpkin seed meal) contribute to a higher nutritional value of crackers in terms of a higher content of protein, unsaturated fatty acids, and minerals. Moreover, crackers from wholemeal flours with a milder taste (maize and millet) are better accepted than those from buckwheat flour, which is characterised by a specific taste. At the same time, sweet potato and spices have a significant influence on the acceptance of the product, as does the addition of sourdough, which has the potential to improve the taste and appearance of the crackers. Unlike total dietary fibre content that obstructs important descriptive sensory properties of crackers such as taste and flavour, soluble fibre content confines undesirable descriptive texture attributes and bitter taste. Nevertheless, soluble fibre and fat content contrast crackers’ hedonic qualities. In the development of products with a high fibre content for IBS sufferers, special attention should be given to the proportion of soluble fibre in total fibre, not only for consumer acceptance, but also for health reasons. Future studies should investigate novel processing techniques in the production of snacks for IBS and NCWS patients, as well as their shelf life.

## Figures and Tables

**Table 1:** Proximate composition of raw materials used in preparation of crackers (g/100 g).

	Chia Seeds	Pumpkin Seed Meal	Flax Seeds	White Maize Flour	Millet Flour	Buckwheat Flour	Rice Protein	Sweet Potato Puree
Proteins	20.0	59.4	23.8	6.3	10.0	10.8	83.0	1.0
Fats	31.0	15.8	26.6	0.9	3.3	2.9	4.5	1.9
Carbohydrates	6.3	nd	9.0	78.0	73.0	70.1	2.9	7.6
Salt	0.05	na	0.1	0.3	0.2	0.1	<0.05	0.03

nd—not detected; na—not available

**Table 2:** Nine gluten-free formulations as the percentage of raw materials.

		F1	F2	F3	F4	F5	F6	F7	F8	F9
Main flours (% of total flour)	Maize	70	70	70	70	22	/	/	/	/
	Proso millet	30	30	30	30	45	50	20	/	85
	Buckwheat	/	/	/	/	33	50	80	100	15
Additional ingredients (% of flour weight)	Water	100	100	73	73	100	80	80	100	75
	Fat spread	20	24	60	60	42	17	17	/	15
	Olive oil	/	/	/	/	/	/	/	25	/
	Rice protein	30	30	36	36	20	30	30	30	10
	Flax seeds	/	/	/	18		12		15	5
	Chia seeds	10	10	18	/	14	/	10	/	
	Pumpkin seed meal	/	/	/	/	20			/	40
	Sweet potato puree	/	/	35	35	/	20	15	30	30
	Salt	1.25	2	2.4	2.4	1.4	0.8	1	1	1
	Spices	/	/	Med. mix 1.2	Chives 2.4	Pepper; wild garlic 0.7; 0.7	Med. mix 0.8	Wild garlic; shallot 0.5; 0.5	Tumeric; dill 0.5; 2	/
	Yeast	/	2	/	/	/	/			/
Soudough (% of dough weight)		20	-	20	20	20	20	20	20	20

Med.mix—Mediterranean seasoning mix

**Table 3:** Selected sensory descriptors and their definition.

Sensory Attribute		Description
Appearance	Colour	Degree of brownness, ranging from light brown to dark brown
	Uniformity of surface	Uniform–non-uniform, smooth–rough
Odour	Overall	Overall intensity of odour
Taste and flavour	Bitter taste	Basic taste produced by caffeine
	Bitter aftertaste	Bitterness after chewing
	Overall	Overall intensity of taste and flavour
Texture in mouth	Hardness	Force applied by the molar teeth to compress the cracker
	Chewiness	Number of chews necessary for food to be swallowed
	Granularity	Sense of particle size and shape (larger particles–higher granularity)
	Dryness	Amount of saliva absorbed by sample crumbs during mastication
	Solubility	Chewing required until the biscuit disintegrates (more chewing–less solubility)
	Teeth adhesiveness	Ability of food to adhere to the teeth when chewed

**Table 4:** Fructan, fibre, and minerals content in raw materials (g/100 g of sample, *n* = 2, expressed on sample dry matter as mean ± standard deviation).

Raw Material	Fructan and Galactooligosaccharides	Total Dietary Fibre	Soluble Fibre	Insoluble Fibre	Minerals (As ash)
Buckwheat flour	nd	6.12 ± 0.53	1.55 ± 0.03	4.57 ± 0.12	3.01 ± 0.00
Millet flour	0.29 ± 0.01	14.90 ± 0.35	8.01 ± 0.14	6.89 ± 0.11	1.26 ± 0.02
White maize flour	0.03 ± 0.06	2.34 ± 0.15	1.09 ± 0.03	1.25 ± 0.03	0.97 ± 0.09
Rice protein	0.14 ± 0.00	3.78 ± 0.69	2.21 ± 0.48	1.57 ± 0.21	1.19 ± 0.07
Chia seeds	0.36 ± 0.00	37.90 ± 0.04	7.26 ± 0.00	30.64 ± 0.00	4.76 ± 0.00
Flax seeds	0.64 ± 0.22	27.88 ± 0.00	7.24 ± 0.00	20.64 ± 0.21	3.85 ± 0.01
Pumpkin seed meal	0.83 ± 0.03	14.80 ± 0.01	6.86 ± 0.02	7.94 ± 0.09	7.49 ± 0.06

nd—not detected

**Table 5:** Energy and nutritive value of the different types of crackers (g/100 g of sample, *n* = 2, expressed on sample dry matter as mean ± standard deviation).

	Cracker F3	Cracker F4	Cracker F5	Cracker F8
Energy (kJ/kcal)	1883/450	1866/446	1820/435	1871/447
Water content	4.86 ± 0.04 ^c^	5.48 ± 0.05 ^b^	4.31 ± 0.04 ^d^	8.02 ± 0.05 ^a^
Minerals (as ash)	2.81 ± 0.01 ^c^	2.91 ± 0.01 ^c^	5.59 ± 0.00 ^b^	6.33 ± 0.00 ^a^
Fats	17.72 ± 0.12 ^a,b^	17.11 ± 0.62 ^b,c^	14.98 ± 0.00 ^c^	19.60 ± 0.10 ^a^
of which saturated	1.37 ± 0.00	1.54 ± 0.00	1.66 ± 0.00	2.71 ± 0.00
Proteins	24.94 ± 0.14 ^b,c^	25.97 ± 0.04 ^a^	26.08 ± 0.04a ^b^	24.02 ± 0.03 ^c^
Carbohydrates	44.00 ± 0.28 ^b^	43.51 ± 0.69 ^b^	44.41 ± 2.67 ^b^	38.83 ± 2.80 ^a^
Dietary fibre	7.49 ± 0.21 ^c^	7.12 ± 0.18 ^d^	8.92 ± 0.58 ^b^	9.53 ± 0.78 ^a^
of which soluble	1.25 ± 0.13 ^d^	1.65 ± 0.08 ^c^	2.06 ± 0.78 ^b^	2.34 ± 0.51 ^a^
Fructan	0.10 ± 0.00 ^a^	0.12 ± 0.00 ^a^	0.12 ± 0.00 ^a^	0.11 ± 0.00 ^a^

Samples within the same row marked with different letters are statistically significantly different (*p* < 0.05)

## Data Availability

The data used to support the findings of this study are included in the article.
